# Proteomic profiling reveals candidate biomarkers of ovarian inactivity in yak via Astral-DIA

**DOI:** 10.3389/fvets.2026.1973877

**Published:** 2026-09-23

**Authors:** Wanshu Wang, Yujun Li, Peng Wang, Min Ran, Yuehuan Zhang, Huizhu Zhang, Yaying Wang, Jiamei Luo, Bingjie Wang, Jian Li, Honghong He

**Affiliations:** 1 Qinghai-Tibetan Plateau Animal Genetic Resource Reservation and Utilization Key Laboratory of Sichuan Province, Southwest Minzu University, Chengdu, Sichuan, China; 2 Key Laboratory of Qinghai-Tibetan Plateau Animal Genetic Resource Reservation and Utilization, Ministry of Education, Chengdu, China; 3 College of Animal Science and Veterinary Medicine, Southwest Minzu University, Chengdu, China; 4 Animal Husbandry Science Institute of Ganzi Tibetan Autonomous Prefecture, Kangding, China

**Keywords:** *Bos grunniens*, inactive ovaries, ovarian function, proteomics, veterinary obstetrics

## Abstract

**Background:**

Inactive ovaries are one of the major causes of low reproductive performance in yaks, but their underlying molecular mechanisms remain unclear.

**Methods:**

In this study, we applied the high‑throughput and high‑sensitivity Astral‑DIA (Data‑Independent Acquisition) technique to identify differentially expressed proteins in ovarian tissues from yaks with inactive ovaries (IO) compared to those in natural estrus (ECG).

**Results:**

A total of 165 highly significant differentially expressed proteins were identified, including 87 upregulated and 78 downregulated proteins. Gene Ontology (GO), EggNOG functional classification, KEGG pathway analysis, and subcellular localization prediction were performed to annotate and characterize these proteins. Protein–protein interaction (PPI) network analysis revealed five representative differential proteins—MAO, FSTL3, HAPLN1, PTGFR, and RDH11—as potential key regulators of ovarian activity. Western blot validation confirmed the consistency of the proteomic data, showing that MAO, FSTL3, and HAPLN1 were significantly upregulated, while PTGFR and RDH11 were downregulated in the IO group.

**Conclusion:**

These findings suggest that these five proteins may be involved in the pathogenesis of ovarian inactivity and could serve as biomarkers for early diagnosis and therapeutic targets. This study provides a theoretical foundation for further research on molecular mechanisms and prevention strategies for inactive ovaries in yaks.

## 1 Introduction

The yak (*Bos grunniens*) is an iconic symbol of Tibet and high altitude (1). It is the only bovine species native to the Qinghai–Tibetan Plateau and adjacent highlands, highly adapted to high-altitude areas with low temperatures, hypoxia, and prolonged food scarcity (2, 3). Yaks play an indispensable socioeconomic role for local people by providing milk and meat, as few other domestic animals can survive in such harsh conditions (4). However, yaks exhibit low fertility. In general, female yaks give birth every other year, bearing one calf every 2 years or two calves every 3 years, and their average annual reproductive rate is less than 60% (5). Under high-altitude grazing conditions, this low reproductive rate constitutes a major bottleneck restricting yak husbandry and productivity (6). Ovarian inactivity is a key non-infectious reproductive disorder in postpartum yaks and is largely aggravated by cold stress and seasonal nutritional deficiencies on the Qinghai–Tibet Plateau (7). A considerable proportion of postpartum yaks fail to resume cyclic ovarian activity within the same breeding season, resulting in delayed estrus recovery, prolonged calving intervals, and further deterioration of herd reproductive performance, thereby imposing substantial economic losses on local pastoral households (6). Nevertheless, most relevant studies focus mainly on dairy cows, and molecular evidence underlying yak ovarian inactivity remains limited.

Inactive ovaries represent an abnormal condition in the estrous cycle of dairy cows, resulting in no follicles on the surface of the ovaries and no corpus luteum (8). In this condition, the ovaries remain in a prolonged dormant and quiescent state; cows do not show estrus, and progression of this disorder can lead to atrophy or sclerosis of the ovaries (9). Inactive ovaries are a major cause of infertility in dairy cows (10). Infertility due to ovarian dysfunction accounts for 26.3% of ovarian diseases, and up to 40% in high-yielding cows (11), which significantly affects cows’ reproductive performance and reduces their productive and economic value.

Most studies on inactive ovaries have focused on blood proteomics and metabolomics in dairy cows, whereas few have addressed inactive ovaries in yaks. Zhao et al. (12) used iTRAQ/LC–MS/MS to explore differential proteins in the blood of dairy cows with inactive ovaries and identified 14 differential proteins closely associated with this condition. Similarly, Bai et al. (13) used GC/MS and LC/MS to explore differential metabolites in the serum of ovarian-inactive cows and identified 30 differential serum metabolites in inactive ovaries. In this study, we used the latest Astral mass spectrometry analyzer, which has a higher acquisition rate than conventional mass spectrometry analyzers and is characterized by three main features: higher throughput, higher sensitivity, and deeper protein coverage (14, 15). Therefore, we applied the Astral-DIA technique to explore differential proteins in yak ovarian tissues, aiming to identify key differential proteins related to inactive ovaries. These findings provide a foundation for establishing an early warning system for the risk of inactive ovaries and for future in-depth study of the occurrence mechanism.

## 2 Materials and methods

### 2.1 Sample collection

We identified the rutting period of yaks by following methods described in the literature (16, 17). Three healthy female yaks were randomly selected from Jialinmu Slaughterhouse (Hongyuan, Sichuan, China, latitude 31°51’N ~ 33°19’N, longitude 101°51′E ~ 103°23′E; mean altitude 3,600 m) to be in the natural estrous cycle and of comparable size. Three healthy female yaks of comparable size and body size in the natural estrous cycle were randomly selected, and three female yaks of comparable size suffering from inactive ovaries were randomly selected by rectal examination according to the criteria for determining inactive ovaries. Immediately after slaughter, ovarian tissues were collected from the yak, observed and confirmed, and then stored in liquid nitrogen.

### 2.2 Criteria for determining inactive ovaries

The ovaries are normal in size and texture, with smooth surfaces and no corpus luteum or follicles; when ovarian atrophy or sclerosis occurs, the ovaries are reduced in size with smooth, firm surfaces (16–19) (see Figure 1).

**Figure 1:**
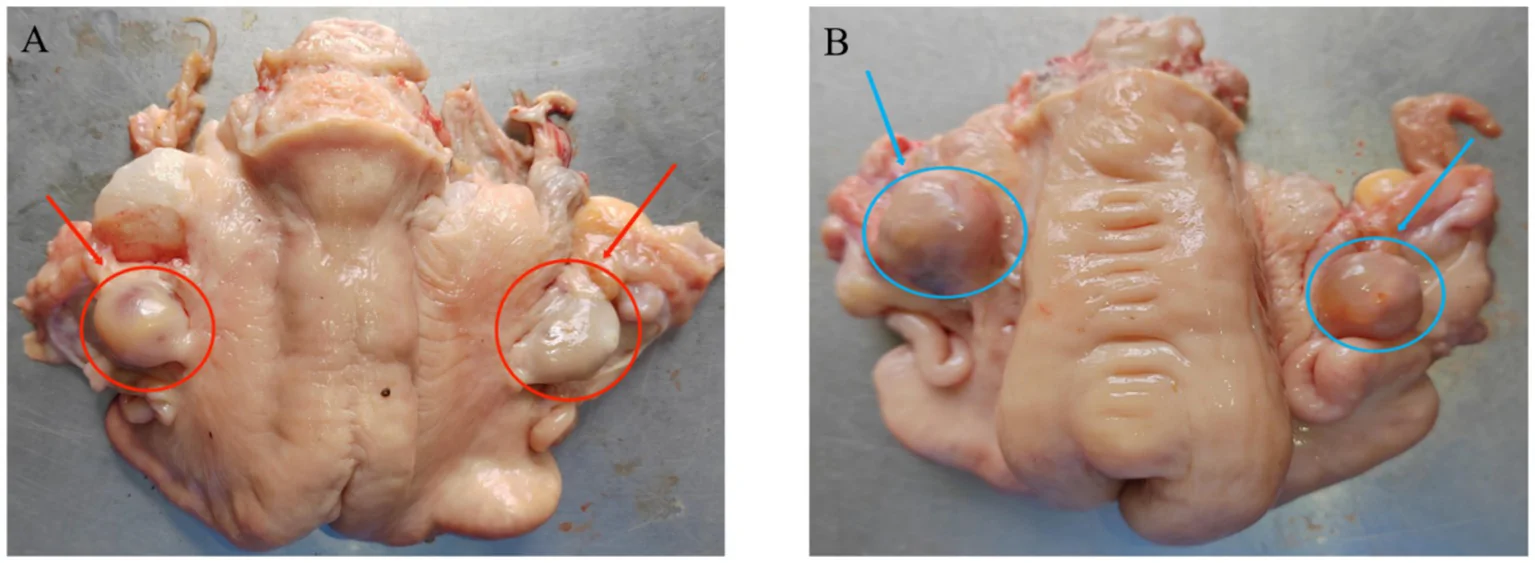
Comparison of anatomical entities of the ovarian tissue in the yaks with inactive ovaries and estrus. Panel **(A)** shows the anatomical morphology of the ovarian tissue in yaks with inactive ovaries; it can be clearly seen that there were no follicles or corpus luteum on the bilateral ovaries, which were smaller in size and had an uneven surface with a firm texture. Panel **(B)** shows the anatomical morphology of ovarian tissue from estrous yaks; it can be clearly seen that there were widely distributed follicles on both ovaries, which were larger in size, with a smooth surface and a soft texture.

### 2.3 Peptide sample preparation

The samples were taken out in the frozen state, and the six samples were divided into two groups: three samples per group (ECG: ECG1, ECG2, and ECG3; IO: IO1, IO2, and IO3). The samples were transferred to a grinding tube, to which lysis buffer (Shanghai National Pharmaceuticals, China) was added. The samples were homogenized three times for 40 s each using a cryomill; incubated on ice for 30 min, during which the samples were vortexed and mixed for 5–10 s at 5-min intervals, and centrifuged at 4 °C for 30 min at 16,000 × *g*, and the supernatants were taken to determine protein concentration using a PierceTM BCA Protein Assay Kit (ThermoFisher Scientific, Waltham, MA, USA).

For each sample, 100 μg of protein solution of the samples to be tested was added; triethylammonium bicarbonate buffer (TEAB, Sigma, USA) was added to achieve a final concentration of TEAB of 100 mM; Tris2-carboxyethyl phosphine (TCEP), Bond-Breaker™ TCEP (ThermoFisher Scientific, USA), was added to a final concentration of 10 mM, and the mixture was incubated at 37 °C for 60 min for disulfide bond reduction; iodoacetamide (IAM, Sigma, USA) was added to a final concentration of 40 mM, and the alkylation reaction proceeded at room temperature and protected from light for 40 min; and centrifuged for 20 min at 10,000 × *g*. The resulting precipitate was centrifuged at 10,000 *g* for 20 min; the sample was fully solubilized with 100 μL of 100 mM TEAB; trypsin (modified trypsin, Promega Corporation, Madison, WI, USA) was added according to the enzyme:protein (m/m) = 1:50, and the enzyme digestion was carried out at 37 °C overnight.

### 2.4 DIA mass spectrometry

After trypsin digestion, an aliquot of the peptide was concentrated using a vacuum centrifuge concentrator. (LNG-T98, Taicang Huamei Biochemistry Instrument Factory, Taicang, Jiangsu, China). The peptides were re-solubilized with 0.1% TFA (Pierce™ Trifluoroacetic Acid, ThermoFisher Scientific, USA); the peptides were desalted and again dried in the vacuum concentrator; and the peptides were quantified using a Peptide Quantification Kit (ThermoFisher Scientific, USA).

The resulting peptides were analyzed using nanoscale liquid chromatography tandem mass spectrometry (VanquishNeo, ThermoFisher, USA) (Astral, ThermoFisher, USA). Equal amounts of peptides were reconstituted in mass spectrometry sample buffer, and the peptides were sampled and separated by a reversed-phase column, uPAC High Throughput column (75 μm × 5.5 cm, ThermoFisher, USA), for 8 min at a flow rate of 1.25–2.5 μL/min. VanquishNeo liquid-phase gradient elution with mobile phase A: 2% acetonitrile (add 0.1% formic acid) and phase B: 80% acetonitrile (add 0.1% formic acid). Gradient elution was performed according to the procedure in Table 1. DIA analysis was then performed in positive ion mode, and the ion source voltage was set to 1.5 kV; Thermo Xcalibur 4.7 software (Thermo, USA) was used for DIA data acquisition.

**Table 1:** Liquid-phase gradient.

Time (min)	B (%)
0	4
0.1	8
1	12.5
1.1	12.6
3.1	22.5
4.6	45
5	99
6.5	stop

Fragment ion peaks were extracted from the DIA raw data using Spectronaut™ 18/DIA-NN software. Six peptides per protein and three fragment ions per peptide were selected for quantitative analysis, with protein FDR ≤ 0.01, peptide FDR ≤ 0.01, peptide confidence ≥ 99%, XIC width ≤ 75 ppm, excluding shared peptides and modified peptides, and the peak areas were summed to obtain the quantitative results. The quantitative results of the identified proteins were normalized by the total peak area ratio and used for differential protein screening and statistics.

### 2.5 Data analysis

The raw files obtained from the mass spectrometry were processed using the UniProt database, and all proteins and protein sequences identified from the mass spectrometer were subjected to full-featured protein annotation searching against the seven major databases (EggNOG, GO, KEGG, NR, Pfam, and UniProt) as well as subcellular localization-related databases. Based on the protein quantitative data, Student’s *t*-test was applied to identify differentially expressed proteins between the IO and ECG groups. The screening thresholds were set as |log2FC| ≥ 1.000 and *p* < 0.01. The proteomic data were deposited in the OMIX database under accession number OMIX018266.

### 2.6 Differential protein verification

Key differential proteins associated with inactive ovaries were screened using proteomics and bioinformatics analyses and validated by Western blot, specific data are shown in Table 2. As with proteomics, we divided the samples into six groups: ECG1, ECG2, ECG3, IO1, IO2, and IO3. The WB experiment was performed with reference to the literature (20–23). Based on the bioinformatics analysis of differential protein function, five representative differential proteins related to inactive ovaries were selected for validation, and each group of samples was loaded at 30 μg. The differential proteins included amine oxidase (MAO), follistatin-related protein 3 (FSTL3), hyaluronan and proteoglycan link protein 1 (HAPLN1), prostaglandin F2-alpha receptor (PTGFR), and retinol dehydrogenase 11 (RDH11). All differential proteins were immunoblotted using respective primary antibodies (bs-11297R MAO from China Bioss; bs-15410R HAPLN1 from China Bioss;bs-16188R FSTL3 from China Bioss; bs-10433R PTGFR from China Bioss, and bs-6214R RDH11 from China Bioss). The secondary antibody was HRP-conjugated Affinipure Goat Anti-Rabbit IgG (H + L) (SA00001-2 Proteintech, USA). The density of the grey bands of differential proteins in the WB was calculated using ImageJ (Rawak Software, Inc., Germany) and subjected it to statistical analysis using the independent samples *t*-test in GraphPad Prism software.

**Table 2:** Differentially expressed proteins between the two groups.

NO.	Accession	Protein name	Log2FC (IO/ECG)	*p*-value Q-(IO/ECG)	Regulate
1	L8IZQ4	Succinyl-CoA:glutarate-CoA transferase	2.53	0.000	Up
2	A0A6B0QY80	MHC class I-like antigen recognition-like domain-containing protein	2.29	0.001	Up
3	A0A6B0R054	Charged multivesicular body protein 3	1.40	0.002	Up
4	A0A6B0R7W6	Calcyphosin	1.75	0.000	Up
5	L8IMK9	VPS35 endosomal protein-sorting factor-like	1.06	0.007	Up
6	A0A6B0REU7	Ras-related protein Rab-32	1.29	0.002	Up
7	A0A6B0RG60	TPR and ankyrin repeat-containing protein 1	1.17	0.001	Up
8	A0A6B0S9J7	N-acylglucosamine 2-epimerase	1.39	0.007	Up
9	A0A6B0REA8	Annexin	2.09	0.002	Up
10	A0A6B0RCX8	MFS domain-containing protein	2.28	0.002	Up
11	A0A6B0QQQ9	Solute carrier family 2, facilitated glucose transporter member 3	1.69	0.000	Up
12	A0A6B0RXZ3	Polymeric immunoglobulin receptor	1.66	0.001	Up
13	L8I018	ADP-ribosylation factor	1.41	0.007	Up
14	L8HTU9	Serpin B4	1.58	0.000	Up
15	A0A6B0QZZ6	Mesothelin	1.60	0.010	Up
16	L8I356	Hyaluronan and proteoglycan link protein 1	3.88	0.003	Up
17	A0A6B0SJ61	HEAT repeat domain-containing protein	2.86	0.009	Up
18	L8IW75	Transmembrane 6 superfamily member1	1.17	0.005	Up
19	A0A6B0RD35	UBC core domain-containing protein	2.29	0.002	Up
20	L8IV10	Ras GTPase-activating-like protein IQGAP2	1.51	0.002	Up
21	A0A6B0RSK6	U1-type domain-containing protein	1.24	0.010	Up
22	A0A6B0QZR0	39S ribosomal protein L20,mitochondrial	1.27	0.003	Up
23	A0A6B0RV45	UBC core domain-containing protein	2.24	0.003	Up
24	A0A6B0S7Q7	Gamma-glutamyltranspeptidase 1	1.51	0.001	Up
25	A0A6B0QUV7	Cnd3 domain-containing protein	1.48	0.007	Up
26	A0A6B0S5T3	MHC-I-like-Ag-recog domain-containing protein	2.29	0.004	Up
27	A0A6B0RQV0	Mab-21 domain-containing protein	1.73	0.007	Up
28	A0A6B0RSV2	DNA topoisomerase 2	1.26	0.006	Up
29	L8I0M0	DDB1- and CUL4-associated factor 11	1.33	0.006	Up
30	A0A6B0RZZ1	FGGY carbohydrate kinase domain-containing protein	1.76	0.008	Up
31	A0A6B0R1P8	E3 ubiquitin-protein ligase	1.21	0.003	Up
32	A0A6B0RV65	Methyltransf-11 domain-containing protein	1.33	0.000	Up
33	A0A6B0R662	Dehydrogenase/reductase 12	1.28	0.005	Up
34	A0A6B0SDJ0	Monoamine Oxidase	5.78	0.001	Up
35	L8IMH3	Unconventional myosin-VI	5.78	0.000	Up
36	A0A6B0RHM7	NHS-like protein 1	5.78	0.000	Up
37	A0A6B0R8E4	Signal-regulatory protein beta-1	5.78	0.004	Up
38	A0A6B0SE88	Secreted seminal-vesicle Ly-6 protein 1-like	5.78	0.002	Up
39	A0A6B0QWN2	TPR-REGION domain-containing protein	5.78	0.006	Up
40	A0A6B0QQ49	Signal transducer and activator of transcription	5.78	0.000	Up
41	A0A6B0QR09	Branched-chain-amino-acid aminotransferase	5.78	0.001	Up
42	A0A6B0RV30	25-hydroxycholesterol 7-Alpha-hydroxylase	5.78	0.008	Up
43	A0A6B0RLR9	Follistatin-related protein 3	5.78	0.000	Up
44	A0A6B0RDE3	Fat storage inducing transmembrane protein 2	5.78	0.000	Up
45	A0A6B0RJS8	Xin actin-binding repeat-containing protein 1	5.78	0.008	Up
46	A0A6B0SCR1	Polyketide_cyc domain-containing protein	5.78	0.002	Up
47	L8HYK0	IF rod domain-containing protein	5.78	0.001	Up
48	L8I4G0	Glycerophosphocholine cholinephosphodiesterase	5.78	0.005	Up
49	L8I937	Vimentin-type intermediate filament-associated coiled-coil protein	5.78	0.000	Up
50	A0A6B0RAQ5	SPATS2	5.78	0.001	Up
51	A0A6B0QTB8	Mitochondrial pyruvate carrier	5.78	0.003	Up
52	A0A6B0RP70	Glutaminyl-peptide cyclotransferase	5.78	0.006	Up
53	A0A6B0R1C6	Rab-GAP TBC domain-containing protein	5.78	0.001	Up
54	A0A6B0S733	Amino acid transporter	5.78	0.005	Up
55	L8HUI6	COX assembly mitochondrial protein	5.78	0.000	Up
56	A0A6B0QSJ9	Ubiquitin carboxyl-terminal hydrolase	5.78	0.000	Up
57	A0A6B0QXF4	HECT-type E3 ubiquitin transferase	5.78	0.004	Up
58	A0A6B0QVP1	GATOR complex protein WDR24	5.78	0.000	Up
59	A0A6B0R0W2	MFS domain-containing protein	5.78	0.003	Up
60	L8IL34	Carboxylic ester hydrolase	5.78	0.006	Up
61	L8I632	Perilipin-2	5.78	0.000	Up
62	A0A6B0R804	Receptor protein serine/threonine kinase	5.78	0.000	Up
63	A0A6B0RF55	CP-type G domain-containing protein	5.78	0.000	Up
64	A0A6B0QSI6	tRNA N6-adenosine threonylcarbamoyltransferase, mitochondrial	5.78	0.009	Up
65	A0A6B0S943	RabBD domain-containing protein	5.78	0.001	UP
66	A0A6B0S818	NFKB inhibitor beta	5.78	0.001	Up
67	L8I4A2	RNA helicase	5.78	0.001	Up
68	A0A6B0RJ40	Tripartite motif-containing protein 16	5.78	0.000	Up
69	L8IXT5	Autophagy-related protein 9	5.78	0.000	Up
70	A0A6B0SAK2	Carboxypeptidase M	5.78	0.001	Up
71	A0A6B0R368	Unconventional myosin-X	5.78	0.000	Up
72	L8IC23	Echinoderm microtubule-associated protein-like 2	5.78	0.004	Up
73	L8J157	Alpha-internexin	5.78	0.001	Up
74	L8ITP4	SH3 domain-binding protein 5	5.78	0.000	Up
75	A0A6B0RDI3	ADP-ribosyl cyclase/cyclic ADP-ribose hydrolase 1	5.78	0.001	Up
76	A0A6B0R6E2	Fidgetin-like protein 1	5.78	0.000	Up
77	A0A6B0SFM5	Zinc knuckle domain-containing protein	5.78	0.004	Up
78	A0A6B0RFM2	CDK5 regulatory subunit associated protein 1	5.78	0.006	Up
79	A0A6B0RXP8	EGF-like domain-containing protein	5.78	0.001	Up
80	A0A6B0S841	UBC core domain-containing protein	5.78	0.006	Up
81	L8I5V1	UV radiation resistance-associated protein	5.78	0.001	Up
82	A0A6B0S0D9	NUC153 domain-containing protein	5.78	0.001	Up
83	L8HN19	Beta-defensin 11	5.78	0.002	Up
84	L8IFQ0	RNA-dependent RNA polymerase	5.78	0.000	Up
85	A0A6B0S6Y0	Protein kinase domain-containing protein	5.78	0.000	Up
86	L8ID24	Lysosomal acid phosphatase	5.78	0.000	Up
87	A0A6B0R242	J domain-containing protein	5.78	0.009	Up
88	A0A6B0RZL6	DEP domain-containing mTOR-interacting protein	−1.09	0.005	Down
89	A0A6B0R696	Carbonic anhydrase 4	−1.08	0.010	Down
90	A0A6B0S1Q2	Lamin	−7.17	0.002	Down
91	L8IYJ3	3-hydroxy-3-methylglutaryl coenzyme A reductase	−3.90	0.000	Down
92	L8IDF6	Protein HP-25-like protein 1	−1.79	0.001	Down
93	L8J297	Nicotinate phosphoribosyltransferase	−1.52	0.003	Down
94	L8I1C5	Cytochrome b-c1 complex subunit 6, mitochondrial	−1.34	0.001	Down
95	L8HSF5	Tripeptidyl-peptidase 2	−1.41	0.008	Down
96	L8IQS3	Synaptoporin	−1.63	0.002	Down
97	L8J050	Transmembrane 9 superfamily member	−1.39	0.007	Down
98	A0A6B0RKK2	Protein HEG homolog 1	−1.38	0.002	Down
99	L8ID48	Exocyst complex component 3-like protein 2	−1.88	0.001	Down
100	A0A6B0RBL6	Alpha-1,2-Mannosidase	−1.16	0.003	Down
101	L8HTU6	Transmembrane protein 176A	−1.54	0.003	Down
102	A0A6B0RV03	Mitochondrial inner membrane protease ATP23	−2.02	0.005	Down
103	A0A6B0RVB7	Acyl-coenzyme A thioesterase 11	−1.42	0.009	Down
104	A0A6B0QX51	Prostaglandin F2-alpha receptor	−16.61	0.000	Down
105	A0A6B0R397	Acyl-CoA dehydrogenase family member 10	−16.61	0.000	Down
106	L8ITF0	Ubiquitin thioesterase OTU1	−16.61	0.003	Down
107	A0A6B0SJG9	Fibrinogen C-terminal domain-containing protein	−16.61	0.001	Down
108	A0A6B0SAA8	Immunoglobulin lambda-1 light chain-like	−16.61	0.000	Down
109	A0A6B0RSI7	Sulfotransferase	−16.61	0.000	Down
110	L8IQG7	MAP7 domain-containing protein 1	−16.61	0.001	Down
111	L8I7Q4	Torsin-1A-interacting protein 1	−16.61	0.000	Down
112	L8I5Z0	BOLA class I histocompatibility antigen, alpha chain BL3-7	−16.61	0.001	Down
113	A0A6B0S3L2	TGS domain-containing protein	−16.61	0.008	Down
114	L8IBS6	Gliomedin	−16.61	0.002	Down
115	L8HSR9	Acyl-CoA dehydrogenase family member9,mitochondrial	−16.61	0.000	Down
116	A0A6B0RW98	Retinoic acid receptor responder protein 2	−16.61	0.002	Down
117	L8ILN6	Protein Mpv17	−16.61	0.005	Down
118	L8HYI1	Retinol dehydrogenase 11	−16.61	0.008	Down
119	A0A6B0RDE0	Bcl-2-like protein 11	−16.61	0.000	Down
120	A0A6B0RHI7	Pept-C1 domain-containing protein	−16.61	0.002	Down
121	L8HRA3	Glutamine--tRNA ligase	−16.61	0.000	Down
122	L8IX13	Cytoskeleton-associated protein 2-like protein	−16.61	0.005	Down
123	L8IYZ6	Mediator of RNA polymerase II transcription subunit 6	−16.61	0.000	Down
124	L8I335	Neurotensin/neuromedin N	−16.61	0.000	Down
125	L8IRQ5	Guanylate-binding protein 6	−16.61	0.003	Down
126	A0A6B0RNE7	Serine aminopeptidase S33 domain-containing protein	−16.61	0.000	Down
127	A0A6B0QTP6	Hemoglobin subunit zeta	−16.61	0.006	Down
128	A0A6B0SCW1	Uncharacterized protein	−16.61	0.000	Down
129	A0A6B0RYZ4	Transmembrane protein 250	−16.61	0.008	Down
130	L8IG49	Protein NDNF	−16.61	0.004	Down
131	A0A6B0R656	Peptidase S1 domain-containing protein	−16.61	0.000	Down
132	A0A6B0RF37	Receptor protein-tyrosine kinase	−16.61	0.008	Down
133	L8I8F3	Inositol 1,4,5-trisphosphate receptor	−16.61	0.000	Down
134	L8HU72	Condensin complex subunit 1	−16.61	0.001	Down
135	A0A6B0RYY1	Serine/threonine-protein kinase	−16.61	0.000	Down
136	A0A6B0S4W4	Rho-related GTP-binding protein RhoU	−16.61	0.002	Down
137	L8HMQ0	Condensin complex subunit 1	−16.61	0.000	Down
138	A0A6B0R5V3	MFS domain-containing protein	−16.61	0.000	Down
139	L8IGG0	Thiopurine S-methyltransferase	−16.61	0.000	Down
140	L8IUL6	ATP synthase subunit s-like protein	−16.61	0.000	Down
141	L8I3S1	ABC-type glutathione-S-conjugate transporter	−16.61	0.001	Down
142	A0A6B0R7J4	Histone-lysine N-methyltransferase SETD1B	−16.61	0.001	Down
143	A0A6B0RWT8	Unconventional myosin-IXa	−16.61	0.002	Down
144	A0A6B0RY89	Protein Shroom2	−16.61	0.004	Down
145	L8IUC3	RB1-inducible coiled-coil protein 1	−16.61	0.000	Down
146	L8HXJ3	Early endosome antigen 1	−16.61	0.000	Down
147	L8J3Q3	Delta-like protein	−16.61	0.009	Down
148	A0A6B0RW40	Tetratricopeptide repeat domain 13	−16.61	0.003	Down
149	A0A6B0QYQ8	PKD domain-containing protein	−16.61	0.005	Down
150	A0A6B0RUM0	Transmembrane and coiled-coil domain-containing protein 4	−16.61	0.007	Down
151	A0A6B0R4K0	YTH domain-containing protein	−16.61	0.000	Down
152	L8IX61	Ig-like domain-containing protein	−16.61	0.000	Down
153	L8I2F0	Conserved oligomeric Golgi complex subunit 1	−16.61	0.000	Down
154	A0A6B0S4I6	Mid1-interacting protein 1	−16.61	0.001	Down
155	A0A6B0S3E1	LRAT domain-containing protein	−16.61	0.001	Down
156	A0A6B0QNZ4	Ligand-dependent nuclear receptor-interacting factor 1	−16.61	0.000	Down
157	A0A6B0R0J1	Reverse transcriptase domain-containing protein	−16.61	0.005	Down
158	A0A6B0RQR3	Histone acetyltransferase	−16.61	0.006	Down
159	L8IQK7	Aryl hydrocarbon receptor	−16.61	0.007	Down
160	A0A6B0QRL0	Synapsin-3	−16.61	0.005	Down
161	L8HQQ0	Leucine--tRNA ligase	−16.61	0.000	Down
162	A0A6B0R3A5	DUF1619 domain-containing protein	−16.61	0.005	Down
163	A0A6B0SBS2	Multidrug resistance-associated protein 4	−16.61	0.001	Down
164	A0A6B0RFV9	Androgen-induced gene 1 protein	−16.61	0.003	Down
165	A0A6B0S381	Cyclin-dependent kinases regulatory subunit	−16.61	0.000	Down

## 3 Results and analysis

### 3.1 Comparative sample analysis

This study aimed to identify representative differentially expressed proteins in the ovarian tissues of yaks with inactive ovaries and explore their potential roles in the onset of ovarian inactivity. A total of six female yaks of comparable body size were randomly selected from Jialinmu Slaughterhouse (Aba Tibetan and Qiang Autonomous Prefecture, Sichuan Province, China). Three yaks in the natural estrus cycle were assigned to the control group, whereas three yaks diagnosed with inactive ovaries via rectal examination were included in the experimental group. Ovarian tissues were harvested and analyzed using Astral-DIA proteomics technology to identify differentially expressed proteins. The ECG cohort refers to the healthy estrous control group (ECG1, ECG2, and ECG3), and the IO cohort refers to the inactive ovary group (IO1, IO2, and IO3).

The Venn diagram of total proteins is shown in Figure 2A. The overlapping region indicates that the number of proteins shared between groups was 7,129, accounting for 97.09% of the total identified proteome. The non-overlapping regions indicate the number of cohort-specific proteins: 104 proteins specific to the ECG group (1.42%) and 110 proteins specific to the IO group (1.5%).

**Figure 2:**
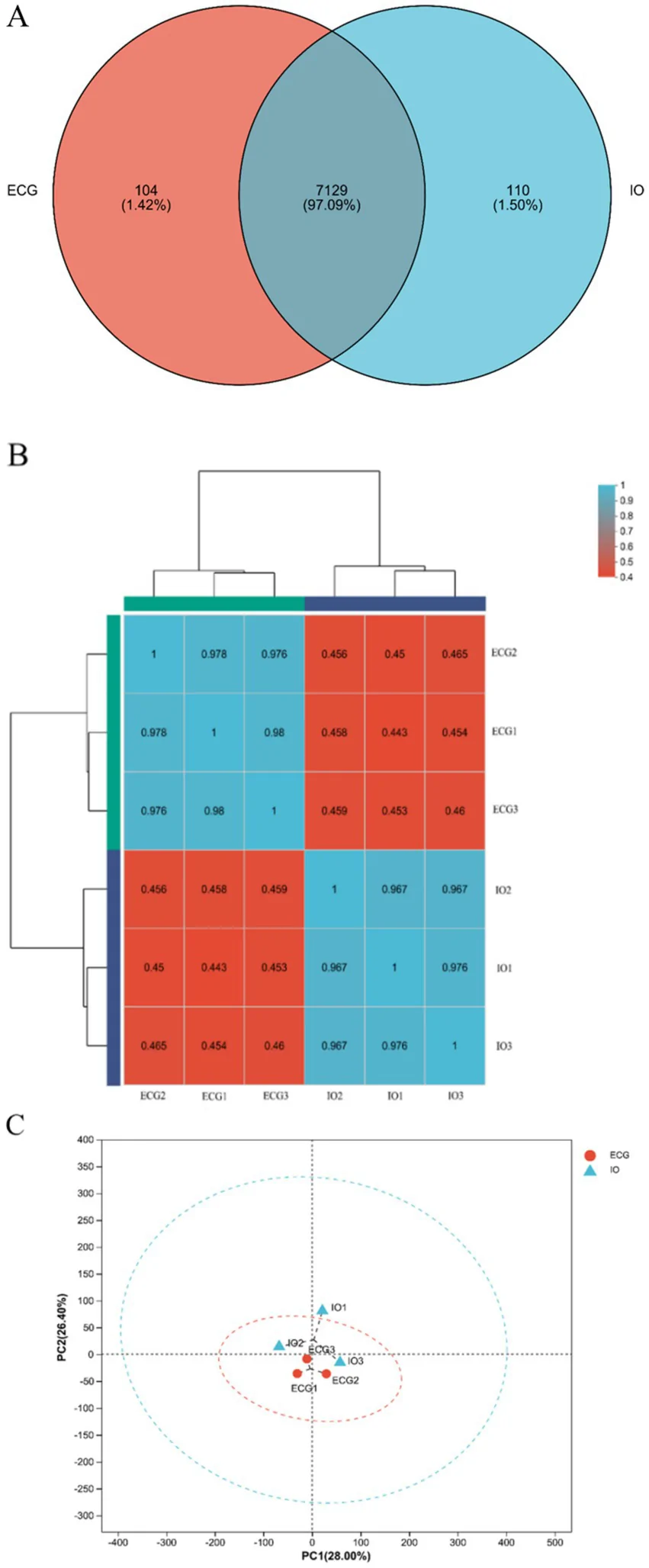
**(A)** Protein Venn plots of ovarian tissue in the yak with inactive ovaries and estrus. **(B)** Sample correlation heat map of ovarian tissue in the yak with inactive ovaries and estrus. **(C)** Principal component analysis of all ovarian tissue proteins in the yak with inactive ovaries and estrus.

The protein expression levels in any two samples were used to calculate the correlation coefficient between each pair of samples, and the sample correlation heatmap is drawn as shown in Figure 2B. Here, each cell represents the correlation between two samples, and different colors denote the magnitude of the correlation coefficient. The results showed that the samples between groups had proteomic differences, whereas the proteomes of the samples within groups were more consistent, indicating high biological replicate reproducibility.

Principal component analysis (PCA) was performed using quantitative protein intensity data from three samples from the inactive ovaries group and three samples from the estrus group, as shown in Figure 2C. The differences between the samples were analyzed through dimensionality reduction, projecting samples onto coordinates on the principal component plane. The distance between the sample points represented the degree of similarity between the samples, where a shorter distance indicated higher similarity between the samples. The results showed that the two groups of samples were distributed in different regions of the two-dimensional space and were clearly differentiated, indicating a distinct trend of separation in the proteomic profiles of the two groups.

### 3.2 Screening and identification of differential proteins

A total of 7,343 proteins were identified in this study. The calculation of fold mean difference was used to screen differentially expressed proteins. To screen highly significant differential proteins, refer to Table 2. As a result, a total of 165 highly significant differentially expressed proteins were identified based on the criteria of (mean fold change > 2, mean fold change < 0.5, |log2FC| ≥ 1.000, *p* < 0.01). Figure 3A shows the bar graphs of differentially expressed proteins in yaks with inactive ovaries compared with those in estrus, of which 87 proteins were upregulated and 78 proteins were downregulated.

**Figure 3:**
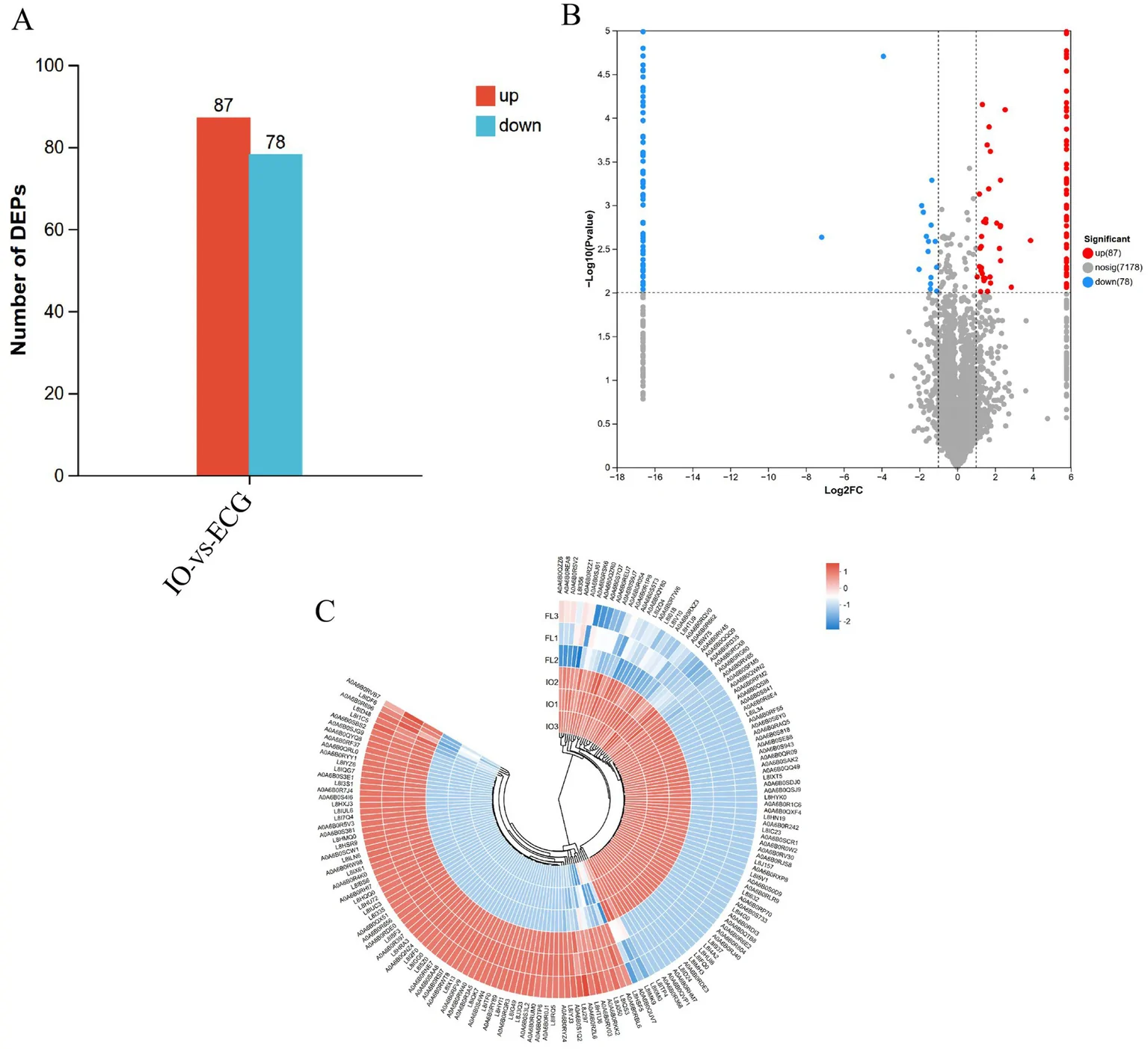
Differentially expressed proteins of ovarian tissue in the yak with inactive ovaries and estrous. **(A)** Number bar charts. **(B)** Volcano plot. **(C)** Heat map.

To further illustrate the differences in protein expression between the inactive ovaries and estrus groups, the 165 differentially expressed proteins were visualized in a volcano plot, as shown in Figure 3B. The results showed that the two groups were clearly distinguished, with good intragroup reproducibility that was consistent with the results of the principal component analysis, confirming that there were significant differences between the two groups at the proteomic level.

Figure 3C presents a heat map showing the expression of 165 differentially expressed proteins across six samples, which visualizes the trend of up- and down-regulation of proteins among different samples. Based on the above analysis, we screened the differentially expressed proteins and reserved them for further functional analysis.

In Figure 3A, the red bars represent upregulated proteins, whereas the blue bars represent downregulated proteins. In Figure 3B, each point represents a specific protein: the points on the left represent downregulated proteins, and the points on the right are the proteins with upregulated expression differences; the further to the left or right, and the higher the point, the more significant the expression differences are. Figure 3C displays a heatmap of the 165 differentially expressed proteins across the six samples (IO1, IO2, IO3, ECG1, ECG2, and ECG3). Rows represent proteins, columns represent samples, and color intensity indicates relative expression levels—red for high expression and blue for low expression. Hierarchical clustering was performed on both proteins and samples, where the proximity of branches in the dendrogram reflects similarity in expression patterns.

### 3.3 GO functional annotation analysis of differentially expressed proteins

The differentially expressed proteins were subjected to Gene Ontology (GO) functional annotation across three categories: biological process (BP), cellular component (CC), and molecular function (MF). The results in Figure 4A show that 387 annotations were assigned to biological processes, mainly associated with cellular processes, metabolic processes, biological regulation, response to stimulus, localization, and development. A total of 199 annotations were annotated to molecular functions, which were mainly involved in binding and catalytic activity. While 174 annotations were annotated to cellular components, primarily associated with cellular anatomical entities. Figure 4B displays the top 20 enriched GO terms after false discovery rate (FDR) correction using the Benjamini–Hochberg (BH) method, including 13 terms for biological processes, six cellular component terms, and one term for molecular function. These annotation and enrichment analyses further extended the molecular characterization of the differentially expressed proteins.

**Figure 4:**
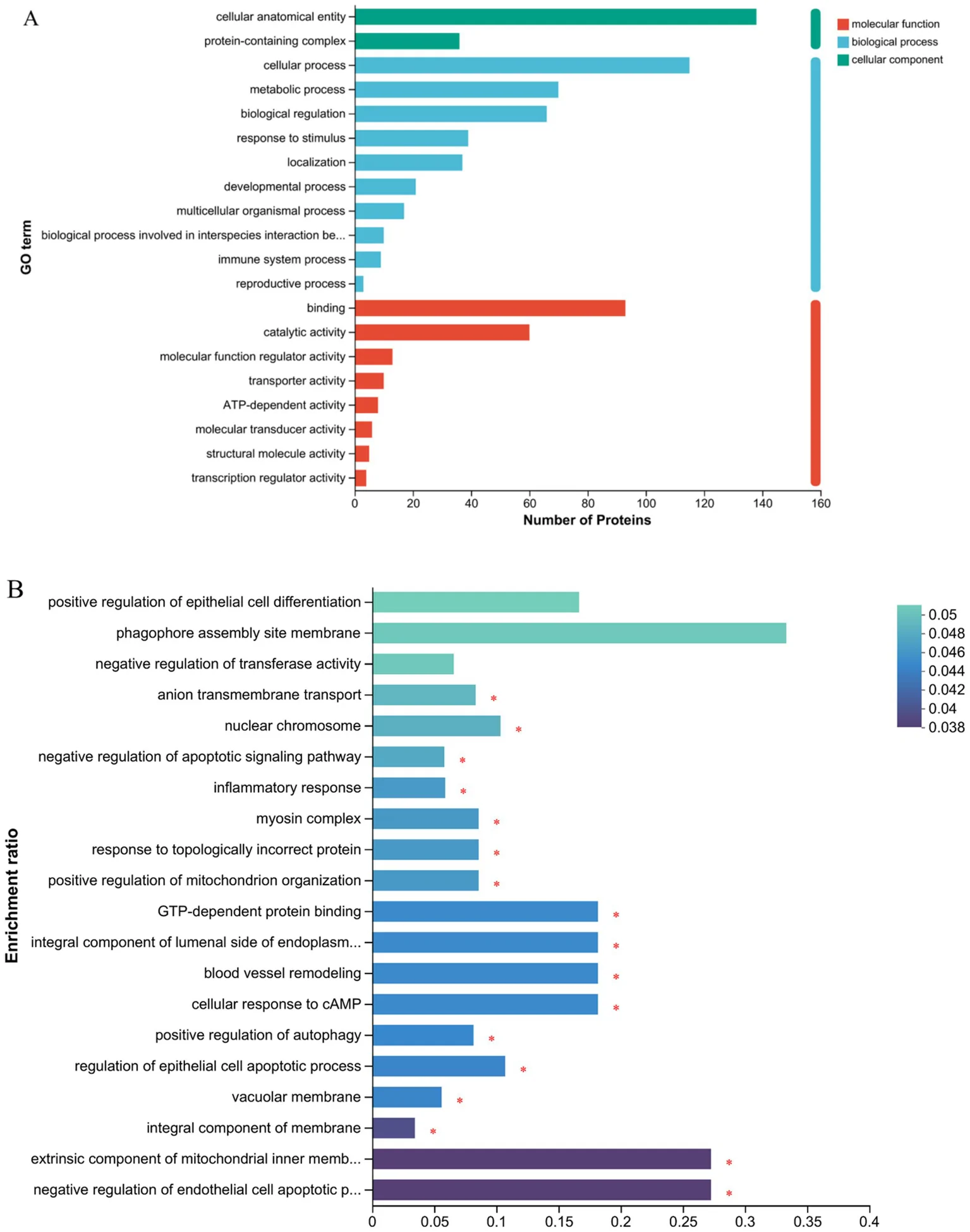
**(A)** GO functional annotation of differentially expressed proteins. **(B)** GO functional enrichment of differentially expressed proteins.

In Figure 4A, the three GO categories are cellular component, biological process, and molecular function. Each bar indicates a GO term, and a higher bar indicates a greater number of proteins annotated to that term; the horizontal coordinate indicates the GO subcategory term, and the vertical coordinate indicates the number of differentially expressed proteins annotated to each term. In Figure 4B, the horizontal coordinate indicates the term name, and the vertical coordinate indicates the enrichment ratio, which refers to the ratio of the number of differentially expressed proteins enriched in the term to the total number of proteins annotated to that GO term. The column color gradient indicates the significance of enrichment, where FDR < 0.05 is marked as asterisk (*).

### 3.4 Prediction of subcellular localization of differentially expressed proteins

Prediction of subcellular localization of the differentially expressed proteins (Figure 5) showed that most proteins were located in the cytoplasm, mitochondria, and nucleus.

**Figure 5:**
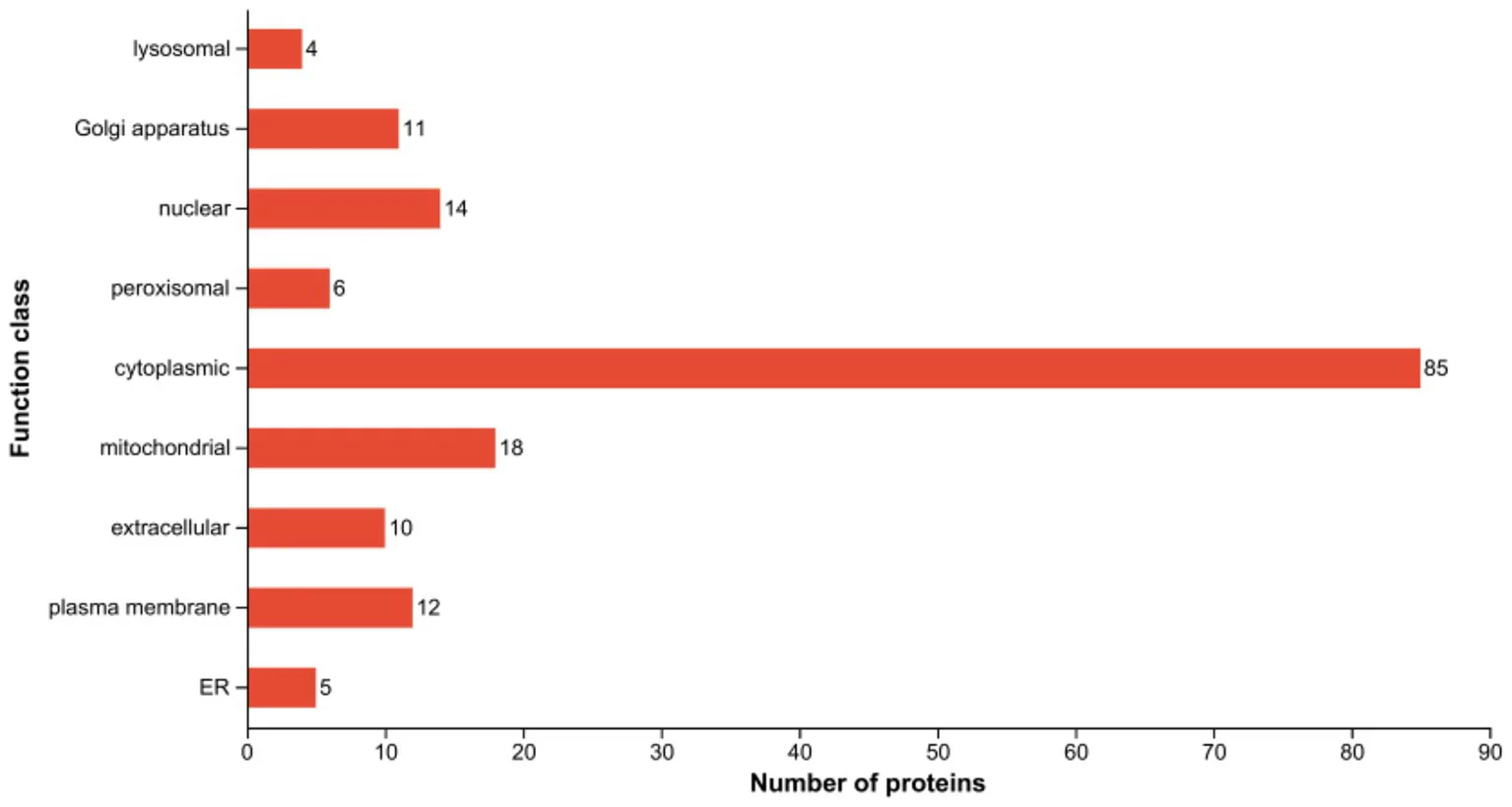
Prediction of subcellular localization of differentially expressed proteins.

### 3.5 EggNOG functional classification of differentially expressed proteins

The EggNOG functional classification of the differentially expressed proteins was performed, and the results are shown in Figure 6, which showed that the differentially expressed proteins were mainly associated with intracellular trafficking, secretion, and vesicular transport, posttranslational modification, protein turnover, and chaperones; and carbohydrate transport and metabolism.

**Figure 6:**
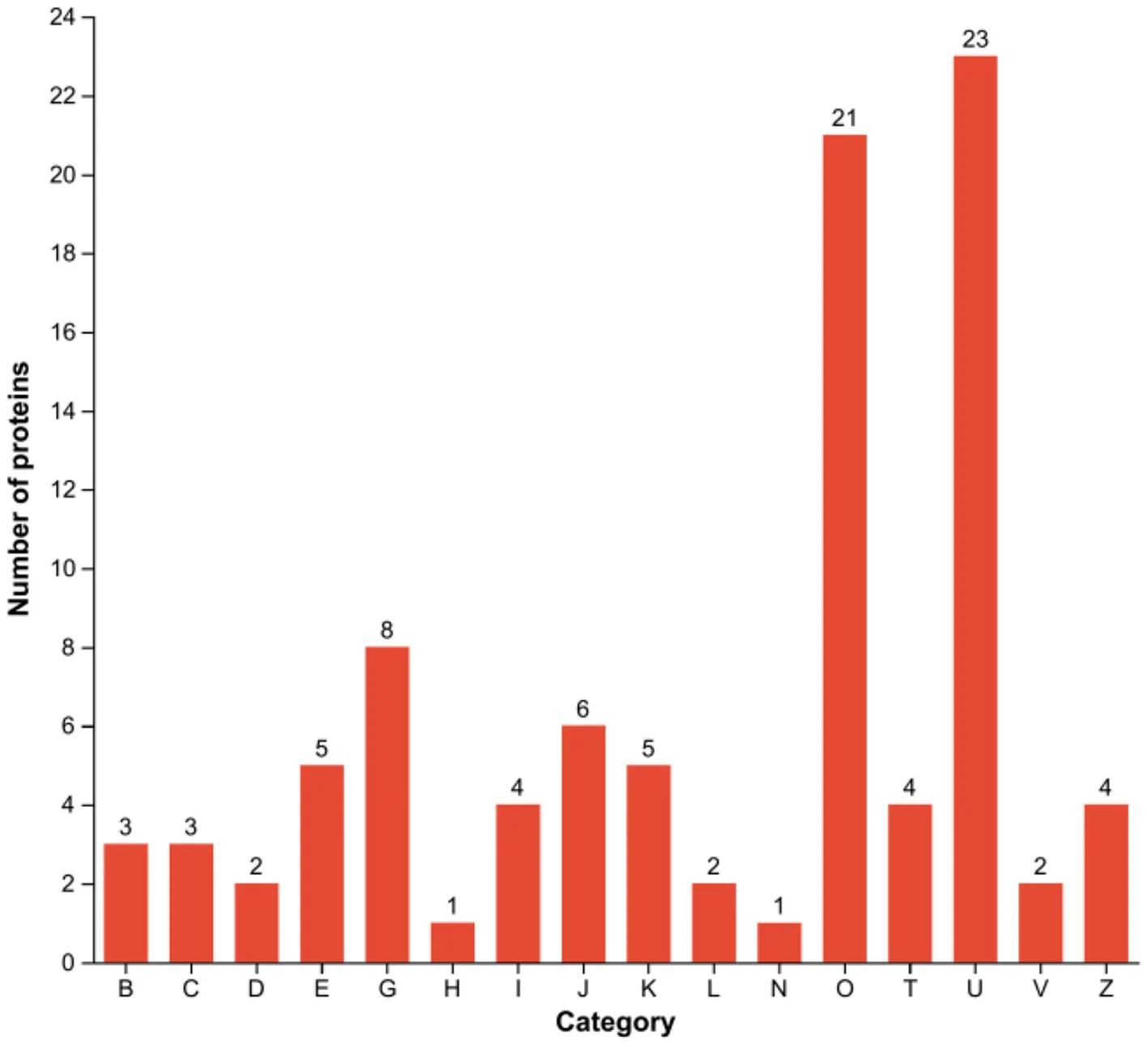
EggNOG functional classification of the differentially expressed proteins. (B) Chromatin structure and dynamics; (C) energy production and conversion; (D) cell cycle control, cell division, chromosome partitioning; (E) amino acid transport and metabolism; (G) carbohydrate transport and metabolism; (H) coenzyme transport and metabolism; (I) lipid transport and metabolism; (J) translation, ribosomal structure, and biogenesis; (K) transcription; (L) replication, recombination, and repair; (N) cell motility; (O) posttranslational modification, protein turnover, chaperones; (T) signal transduction mechanisms; (U) intracellular trafficking, secretion, and vesicular transport; (V) defense mechanisms; and (Z) cytoskeleton.

As shown in Figure 6, the EggNOG functional classification further confirmed that the majority of the differentially expressed proteins were enriched in intracellular trafficking, secretion, and vesicular transport, posttranslational modification, protein turnover, and chaperones; and carbohydrate transport and metabolism, highlighting their potential roles in ovarian physiological processes.

### 3.6 KEGG pathway enrichment of differentially expressed proteins

Differentially expressed proteins were further evaluated using KEGG pathway enrichment analysis. A total of 148 pathways were identified, and the top 20 pathways were visualized in a bubble plot (Figure 7). The enriched KEGG pathways were predominantly associated with bile secretion, type I diabetes mellitus, D-amino acid metabolism, and valine, leucine, and isoleucine biosynthesis.

**Figure 7:**
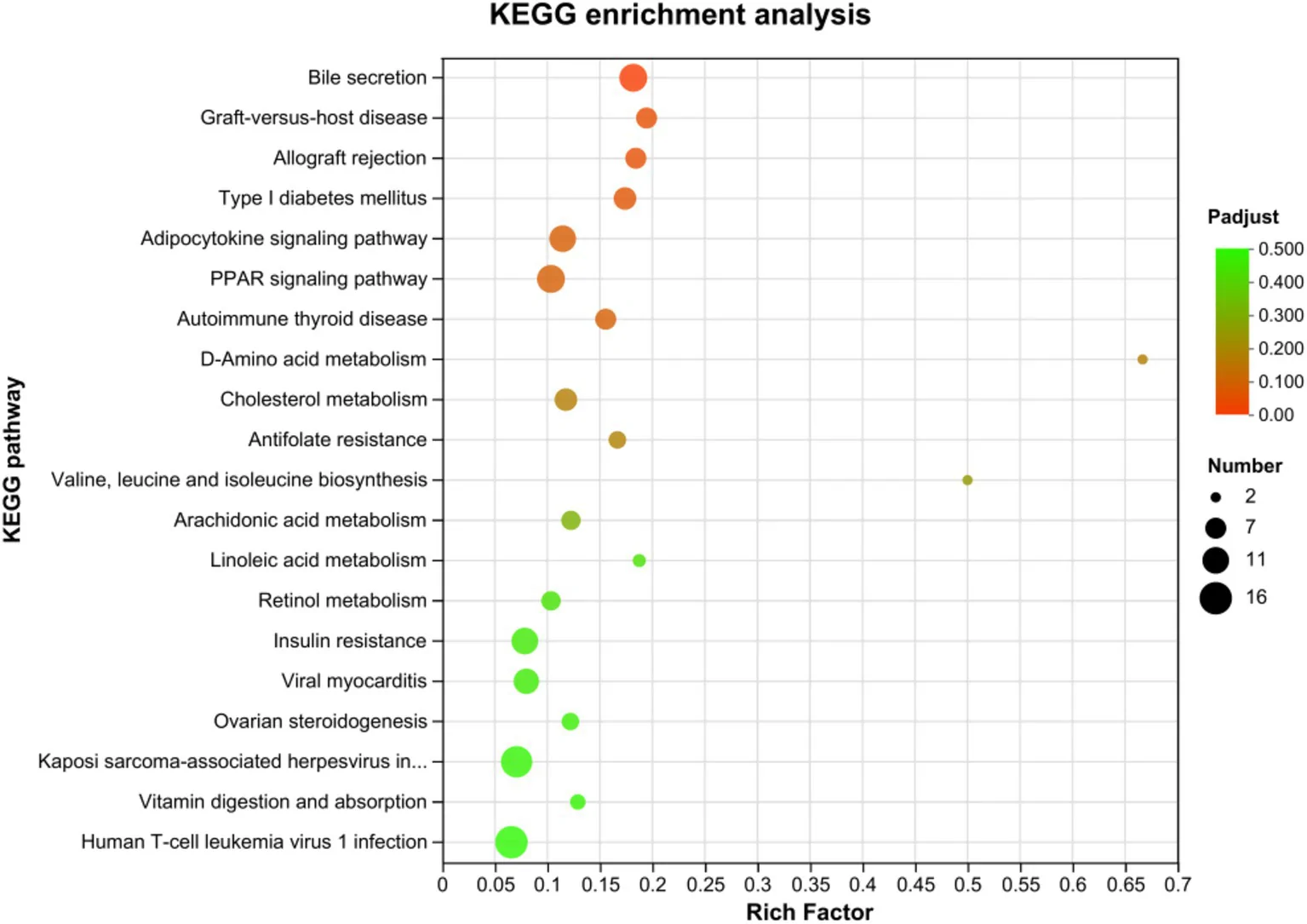
Kyoto encyclopedia of genes and genomes (KEGG) pathway enrichment of differentially expressed proteins in yaks with inactive ovaries compared with those in estrus. The horizontal coordinate represents the enrichment ratio, which refers to the ratio of the number of differentially expressed proteins to the total background number in the pathway; a higher ratio indicates a greater degree of enrichment. The color gradient indicates the statistical significance of enrichment, with darker colors indicating the higher the degree of enrichment, and the size of the bubbles is the number of enriched proteins.

### 3.7 Differential protein interaction network analysis

Prediction of protein–protein interaction (PPI) networks of the differentially expressed proteins was conducted using STRING 12.0. In Figure 8A, each node indicates a protein, and dotted lines indicate predicted interactions between two proteins. The size of each node reflects its degree of connectivity. Larger nodes represent proteins with more interactions, suggesting greater centrality and potential functional importance in the network. The results showed that hub proteins exhibiting high connectivity across the network included MAO, AOC1, COQ10, FSTL3, FSTL5, SPATS2, ATG9, HAPLN1, PTGFR, ACAD10, TIG2, RDH11, and HBAZ. Among these, the key upregulated proteins that were more critical were MAO, FSTL3, and HAPLN1, whereas PTGFR and RDH11 were key downregulated proteins, based on expression levels and network centrality; these five proteins were clustered using STRING 12.0, and the results are shown in Figures 8B–F.

**Figure 8:**
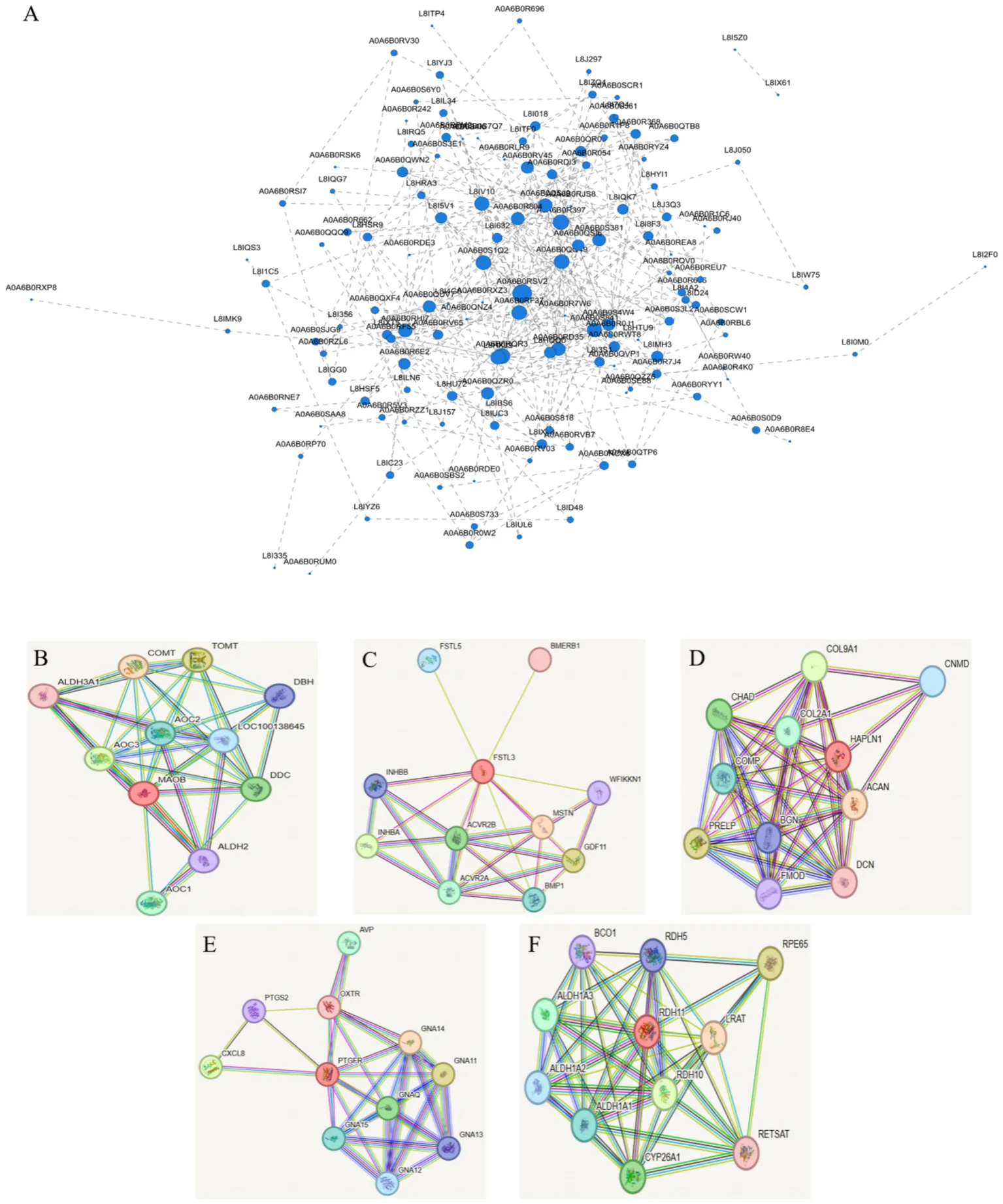
PPI analysis of ovarian tissue in the yak with inactive ovaries and estrus. The nodes in panel **(A)** represent proteins, and the sequence numbers on the nodes are the corresponding IDs of the proteins in the database; panel **(B)** shows the results of MAO clustering; panel **(C)** shows the results of FSTL3 clustering; panel **(D)** shows the results of HAPLN1 clustering; panel **(E)** shows the results of PTGFR clustering; panel **(F)** shows the results of RDH11 clustering.

### 3.8 Differential protein validation results

Five representative differentially expressed proteins (MAO, HAPLN1, FSTL3, PTGFR and RDH11) associated with inactive ovaries identified from proteomic analyses were validated by Western blotting and band intensity quantification. The results of gray-value analysis are shown in Figure 9. The relative expression trends of these five proteins were consistent with those observed in the proteomic data between the inactive ovaries and estrus groups.

**Figure 9:**
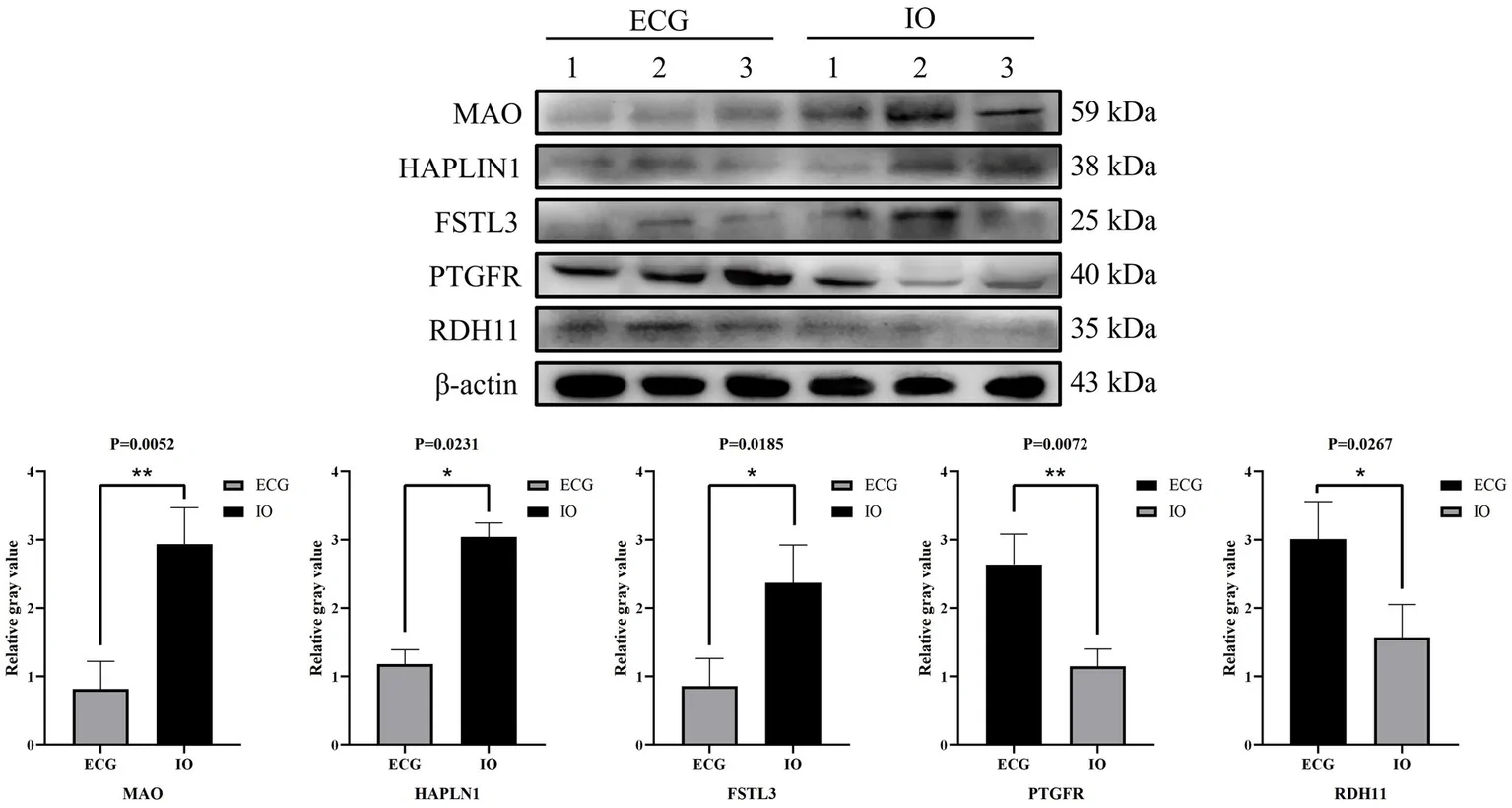
Western blotting and gray-value analysis for MAO, HAPLN1, FSTL3, PTGFR, and RDH11. ECG refers to samples from the healthy normal estrous control group: ECG1, ECG2, ECG3; IO refers to samples from the inactive ovary group: IO1; IO2; IO3. *p < 0.05; **p < 0.01.

## 4 Discussion

In this study, the Astral-DIA technique was used to profile proteomic differences in ovarian tissues between yaks with inactive ovaries and estrous yaks. Subsequent Western blotting validation of five candidate proteins showed expression trends consistent with those in our proteomic dataset, supporting the reliability of our differentially expressed protein screening. These hub molecules are predicted to participate in multiple biological processes, including extracellular-matrix remodeling, steroid metabolism, and prostaglandin-related signaling. Each hub protein may exert unique functions during ovarian dysfunction and will be interpreted separately below. Notably, these proteins do not function in isolation and may act in concert to modulate follicular status.

Functional enrichment analyses were performed for these differentially expressed proteins. Among the enriched KEGG pathways, some terms, such as bile secretion and type I diabetes mellitus, showed limited direct connections with ovarian physiological functions. These signals may have originated from the background noise in proteomic profiling or may have represented secondary systemic metabolic perturbations induced by ovarian inactivity itself; therefore, we did not further expand on them here. Instead, we focused our biological interpretation on ovarian steroidogenesis, which is closely associated with follicular development and ovarian function. Dysregulation of ovarian steroidogenesis can disturb hormone synthesis, block follicular activation, and contribute to the occurrence of ovarian quiescence in yaks.

Monoamine oxidase (MAO) is an enzyme in the oxidoreductase family, which is widely present in both bacteria and humans (24). It plays a central role in the metabolism of biogenic amines and is considered a biological regulator, particularly in regulating cell growth and differentiation (25). Although research on MAO in yaks is limited, studies in other species have provided relevant insights. It was reported that Sun et al. (26) analyzed the transcriptome of goose pre-grade follicles by high-throughput sequencing and found that MAO may be involved in the follicular atresia process. Another study found that injecting MAO inhibitors into the uterus of pregnant rats severely interfered with delivery (27). Furthermore, recent studies have found that injecting fluoxetine into mice interrupts the estrous cycle, with a concomitant increase in MAO expression (28). Taken together, these findings suggest that MAO influences follicular development and may be closely related to inactive ovaries. However, the specific molecular mechanism requires further investigation. Besides MAO-mediated metabolic regulation, extracellular-matrix-related factors also participate in maintaining ovarian homeostasis, among which FSTL3 warrants further consideration.

Follistatin (FS) is an important physiological regulator of activin and other TGF-beta superfamily members. Follistatin-related protein 3 (FSTL3) shares significant structural and functional homology with FS (29), it also plays a role in regulating apoptosis. Overexpression of FSTL3 has been reported to result in ovaries with fewer sinus follicles and significant follicular atresia, ultimately affecting normal gonadal development (30). In addition, recent studies have shown that FSTL3 interferes with ovarian hormone secretion, altering the concentration of estrogen (E2) and progesterone (P4) (31). In conjunction with the results of this experiment, it seems that FSTL3 may play an important role throughout follicular development, and the level of FSTL3 may serve as a marker for determining the onset of inactive ovaries in the future. Consistent with FSTL3, HAPLN1 is also closely associated with ovarian extracellular matrix remodeling.

Hyaluronic acid and proteoglycan link protein 1 (HAPLN1) non-covalently links hyaluronic acid (HA) and proteoglycans (PG), playing a key role in the formation and stabilization of HAPLN1-containing aggregates as a major component of the extracellular matrix (32). Previous studies have reported that treating rat estrus granulosa cells with HAPLN1 improves the viability of cultured granulosa cells and reduces the number of apoptotic cells, whereas knockdown of HAPLN1 expression decreases granulosa cell viability, suggesting that HAPLN1 promotes the survival of ovulatory granulosa cells, thereby facilitating their differentiation into luteal cells (33). Based on the results of the present study, the functional role of HAPLN1 in yaks may be different from that in rats, and HAPLN1 in yaks may influence the onset of inactive ovaries, while the exact mechanism needs to be further investigated. Apart from extracellular matrix modification, prostaglandin-receptor-mediated signaling is another critical pathway affecting ovarian cyclicity, represented by PTGFR in our dataset.

The prostaglandin F2-alpha receptor (PTGFR) is one of the major prostaglandins secreted by the endometrium and plays an important role in regulating endometrial cell viability, apoptosis, and cell cycle. PTGFR has been reported to participate in ovulation and luteinization in dairy cows, thereby affecting follicular development (34). It has been shown that PTGFR promotes growth factor expression by activating the PKC signaling pathway in bovine endometrial epithelial cells (35). Furthermore, recent studies have demonstrated that embryo development in pigs can be promoted by regulating PTGFR expression (36). In our study, the protein expression of PTGFR was significantly higher in the estrous control group than in the inactive ovaries group, suggesting that follicular non-development after the onset of inactive ovaries may be inextricably linked to PGF-2 alpha hormone, and the presence or absence of PGF-2alpha hormone may be used as a future marker for determining the onset of inactive ovaries. Beyond prostaglandin signaling, retinol metabolism mediated by RDH11 is also indispensable for normal follicular development.

Retinol is a fat-soluble nutrient indispensable for mammalian gestation (37). Retinoic acid is the active, oxidized metabolite of retinol (12), deficiency or excess of retinol and retinoic acid can lead to abnormal development of embryos and placental structures. Embryos are unable to synthesize retinol and rely heavily on the maternal supply of retinol itself or its precursors (retinyl esters or carotenoids). In addition, retinoic acid influences FSH receptor and LH receptor levels, the acquisition of which is essential for follicular development, oocyte maturation, ovulation, and luteinization. In this study, retinol dehydrogenase 11 (RDH11), a key protein involved retinol metabolism, the protein expression of RDH11 was expressed at significantly higher levels in the estrous control group than in the inactive ovary group, suggesting that disruption of retinol metabolism may be associated with the development of inactive ovaries. However, the underlying mechanisms require further investigation.

Taken together, although MAO, FSTL3, HAPLN1, PTGFR, and RDH11 perform different biological functions, they are functionally interrelated. MAO is involved in biogenic amine metabolism; FSTL3 and HAPLN1 jointly modulate ovarian extracellular-matrix organization; PTGFR transduces prostaglandin signals for follicle maturation and ovulation; and RDH11 participates in retinol metabolism during folliculogenesis. The dysregulation of these proteins may disturb the coordinated microenvironment within the yak ovary, ultimately hampering follicular activation and leading to ovarian inactivity. Our current data only reflect correlative relationships, and further functional experiments are required to dissect their precise interplay.

In this study, Astral-DIA was applied for the first time to identify differentially expressed proteins in yak ovarian tissues from animals with inactive ovaries and those in estrus. A total of 165 differentially expressed proteins were identified. Among them, five representative differentially expressed proteins associated with the inactive ovaries group were selected, in which MAO, HAPLN1, and FSTL3 were upregulated, and PTGFR and RDH11 were downregulated in the IO group, and the Western blot experiments verified that the results were consistent with proteomics. These findings suggest that these five proteins are closely associated with inactive ovaries in yaks and may serve as markers for early diagnosis. Overall, this study lays a foundation for further research into the underlying mechanisms and potential prevention strategies for inactive ovaries in yaks.

## 5 Conclusion

In this study, Astral-DIA proteomics was used to identify 165 differentially expressed proteins in ovarian tissues from yaks with inactive ovaries and estrous yaks. Bioinformatic and PPI network analyses screened five hub proteins: MAO, FSTL3, HAPLN1, PTGFR, and RDH11. Western blotting verification confirmed that MAO, FSTL3, and HAPLN1 were upregulated, while PTGFR and RDH11 were downregulated in the inactive ovaries, consistent with the proteomic data. These key proteins may contribute to the pathogenesis of yak ovarian inactivity and may serve as candidate biomarkers. This study provides theoretical support for exploring the molecular mechanisms underlying yak inactive ovaries. Further research is needed to clarify their exact regulatory functions.

## Data Availability

The datasets presented in this study can be found in online repositories. The names of the repository/repositories and accession number(s) can be found below: https://ngdc.cncb.ac.cn/omix, OMIX018266.
